# To Protect or to Kill? Environmental Contingent Self-Worth Moderates Death Prime Effects on Animal-Based Attitudes

**DOI:** 10.1177/01461672231160652

**Published:** 2023-03-21

**Authors:** Samuel Fairlamb, Andrada-Elena Stan, Katinka Lovas

**Affiliations:** 1 Royal Holloway, University of London, Egham, UK

**Keywords:** terror management, mortality salience, human–animal relations, violence, environmental self-worth

## Abstract

Lifshin et al. found that death primes increased support for killing animals, suggesting that the killing of animals serves a terror management function. The present research adds to this by suggesting that protecting animals can also serve a terror management function when people see such behaviors as culturally valuable. In three studies (*N* = 765), environmental contingent self-worth (ECSW) moderated the effect of death primes on attitudes toward animals. Attitudes toward animals also mediated the effect of a death prime on increased power-based invulnerability for those with low ECSW and decreased power-based invulnerability for those with high ECSW (Study 3). Finally, we found little support that death primes influenced beliefs regarding human–animal superiority (Study 1 and 2) or similarity (Study 2). Our findings therefore provide partial support for past terror management research and further the understanding regarding how to promote more benevolent human–animal relations.

## Introduction

Every day, humans kill large numbers of animals and destroy their natural habitats. Lifshin et al. (2017) hypothesized that, in part, people’s willingness to kill animals might have a psychological basis. Utilizing insights from terror management theory (TMT; Greenberg et al., 1986), they proposed that the awareness of death may motivate support for killing animals as a function of making people feel superior to animals and elevate themselves above their creatureliness status. In five experiments, they showed that death primes increased people’s support for killing animals. Perhaps surprisingly, they found that attitudes toward animal rights and other psychological constructs (e.g., political orientation) did not moderate these effects.^1^

While we agree that existential concerns might motivate a tendency to support the killing of animals, we find it unlikely that such an effect would be monolithic. For example, there are hundreds of animal welfare charities, organizations, and conservations dedicated to protecting the health, safety, and well-being of animals. Similarly, veganism has increased over the last few years (Wunsch, 2021), with ethical reasons as a common decision behind this choice (Wunsch, 2020). In short, there are numerous examples demonstrating that people see the need to nurture, care for, and protect animals. From a terror management perspective, while the awareness of death might motivate little regard for animal life, if one’s worldview promotes environmental concerns as a way to attain self-esteem, then death primes should promote pro-social attitudes toward animals. We report three studies consistent with this claim.

### Terror Management Theory

TMT (Greenberg et al., 1986) posits that the desire to survive, juxtaposed with the awareness that death is inevitable, produces the potential for debilitating existential anxiety. To manage these death-related anxieties, people invest in, and maintain, cultural worldviews that provide a sense that life has meaning. These worldviews also provide standards of conduct that if lived up to, or exceeded, can offer a sense of self-esteem that transcends one’s mortal, physical self.

Most studies examining TMT claims, including Lifshin et al. (2017), have utilized the mortality salience hypothesis (Burke et al., 2010). This proposes that if psychological structures (i.e., worldviews and self-esteem) manage death-related anxieties, then reminders of death should increase the need for those psychological structures and lead people to behave in accordance with their cultural beliefs. In line with this, hundreds of studies have supported the many ways that different people, from diverse cultures, go about managing death-related concerns (for a review see Pyszczynski et al., 2015).

Because TMT posits that humans hold deep-rooted concerns about their finitude, it argues that people seek to dissociate themselves from animals and nature which are a reminder of their corporeality (e.g., Goldenberg et al., 2000). For example, mortality salience increases people’s preference for essays that suggest humans are different from animals (e.g., Goldenberg et al., 2001). Mortality salience can also increase negative attitudes toward animals (e.g., Beatson & Halloran, 2007). This line of reasoning formed the basis for the prediction that killing animals might serve a terror management function, as it allows people to feel superior to animals and deny one’s own creature-like status (for a more thorough review see Lifshin et al., 2017). In addition, Lifshin et al. (2017) suggested that beliefs regarding human differences and superiority to animals are a fundamental component to almost all worldviews, so the effect of death primes increasing support for killing may not be moderated by attitudes toward animal rights (ATAR). Their studies supported this claim.

### Environmental Contingent Self-Worth

We agree that the awareness of death may psychologically drive humans to separate themselves from nature as well as show little regard for animal life. Yet, we find it hard to imagine that there would not be some circumstances where people may also engage in pro-animal pursuits and believe that protecting animals is a culturally valuable and significant endeavor. As we noted earlier, there are several examples regarding the many ways in which humans from various cultures can show prosocial attitudes toward animals and a need to nurture the environment. Indeed, research has shown that cultural norms play an important role in legitimizing support for killing animals (e.g., Bègue & Vezirian, 2021; Dhont & Hodson, 2014). To be clear, Lifshin and colleagues (2017) did acknowledge the possibility that particular worldview beliefs might moderate their effects but failed to identify this in their studies.

One reason Lifshin and colleagues (2017) may not have found a moderating effect is because of how they opted to tap into the cultural importance to protect animals. The authors measured ATAR which was the extent to which people felt that animal rights were personally important to them. One possibility is that people might fluctuate in the extent to which animal rights are important for reasons other than just terror management ones. In addition, people might identify with animal rights for differing ideological reasons that may subtly influence the ways in which people value and express the importance of animal rights (e.g., Galvin & Herzog, 1992; Wuensch et al., 2002).

In this study, we examine the extent to which people see their self-worth as contingent on their environmental actions (environmental contingencies of self-worth, ECSW; Brook, 2005). We suspect that this would be a stronger moderator to death prime effects on people’s support toward protecting and/or killing animals. This is because ECSW directly taps into whether people see environmental pursuits as a source of self-esteem, which according to TMT is a primary way in which people manage concerns about death (Greenberg et al., 1986). Consistent with this proposition, Vess and Arndt (2008) found that mortality salience increased people’s environmental concern when they had high ECSW. Although this provides good support for our claims, their study examined environmental concerns rather than animal-related attitudes. However, given that environmentalists hold values that concern conserving and protecting natural resources, ecosystems, and wildlife (Dietz et al., 2005), and pro-environmentalism is conceptually related with positive animal attitudes (e.g., Binngießer & Randler, 2015), it should follow that death primes should increase support for animal protection and decrease their support for killing animals for those with high ECSW.

### The Role of Human–Animal Beliefs

Lifshin and colleagues (2017) argued that killing animals may help people feel superior to animals and deny their creature-like status. Supporting this, they found that death primes increased support for killing animals, which in turn increased a sense of power-based invulnerability (Study 5). Despite this evidence, some questions about the role of human–animal beliefs remain. If mortality salience increases a desire to see humans as superior and distinct from animals (e.g., Goldenberg et al., 2001), then presumably these beliefs may be a key mediator in the mortality salience—animal attitude process. We sought to examine this possibility, and whether ECSW would moderate this effect. We envisaged two possibilities.

One possibility might be that, regardless of environmental contingencies, people hold a desire to feel superior to animals as it serves a valuable terror management function. However, environmental contingencies may direct this sense of superiority into positive orientations toward animals. Stated differently, superiority may fuel negative attitudes toward animals with low environmental contingencies but may fuel positive attitudes for those with high environmental contingencies. Some evidence suggests that animal activists view it as an obligation and duty of humankind to use their powers to protect nature and animals (e.g., Bunderson & Thompson, 2009). Thus, people may see their superior status over animals as a call to action to protect them. Potentially consistent with this proposition, among pet owners, who presumably hold positive predispositions toward animals, mortality salience increases positive evaluations of pets when human uniqueness is emphasized, but this effect is reversed when human–animal similarities are emphasized (Beatson et al., 2009). Thus, one interpretation of this finding is that their positive attitudes toward animals were contingent on maintaining a sense of human superiority that emphasizing the similarities between humans and animals threatened.

However, it is worth noting that despite evidence supporting the idea that people prefer to feel that they are superior and different, from animals, a replication of the Goldenberg et al.’s (2001) study did not find support for this claim (Rodríguez-Ferreiro et al., 2019). One possibility is that the need to feel superior, and different, to animals is limited to specific worldviews that differentiates the participants in these two studies. In relation to our research, perhaps environmental contingencies are incompatible with these beliefs. This might attenuate the tendency for people to believe that they are superior and/or different from animals after mortality salience. Indeed, evidence suggests that the more likely people perceive humans and animals to be similar, the more likely they are to support animal rights (e.g., Kellert, 1980).

### Overview of the Present Research

In sum, our primary goal of this research was to examine whether ECSW would moderate the effect of death primes on the support for protecting animals, as well as killing them. We conducted three studies to assess this claim. The studies were not pre-registered. We report all manipulations, measures, and exclusions in these studies. In each study, we measured support for protecting animals, as well as a measure of support for killing animals utilized in Lifshin et al. (2017, Study 1 and 2). Principal components analyses (varimax rotation) supported these as separate factors^2^ suggesting we tapped into two separate constructs regarding support for animals. Our central prediction was that death primes would increase support for killing and decrease support for protecting, animals for those with low ECSW. This effect would be reversed at high levels of ECSW.

In addition, we also examined the effect of death primes on increasing human superiority over animals (Studies 1 and 2), decreasing perceived human–animal similarity (Study 2), and increasing a sense of power-based invulnerability (Study 3). As outlined, we kept open our prediction regarding whether ECSW would moderate these effects as we saw both possibilities as potentially consistent with the theory and past research.

## Study 1

### Participants

All data and materials pertaining to these studies have been made available at: https://osf.io/fz5ua/. We recruited participants via social media and online forums (e.g., Facebook; Reddit sample size) and sought to collect as much as possible by a specified end date. No compensation was provided. In total, 289 participants initiated the study, but only 235 completed it. We excluded 38 participants: (a) 16 did not complete the experimental manipulation, the delay materials or the dependent measures; (b) three suggested we should not use their data as it were unreliable; (c) one expressed knowledge of the real study aim; and (d) 18 took longer than 30 min to complete the study suggesting they did not complete the study in one sitting and the effects of the priming manipulation may have dissipated (average completion time = 11.9 minutes, *SD* = 5.4). This left a final sample of 197 participants (*M*_age_ = 27.7, *SD*_age_ = 11.3) with 130 females, 57 males, and 10 who identified as non-binary. Post hoc sensitivity analyses suggested we had 90% power to detect a small-medium interaction effect f^2^ = .05. Participants were randomly allocated to the control (*n* = 95) or death prime (n = 102). Ethical approval was provided by the university.

### Materials and Procedure

The study was pitched as an examination of personality and environmental attitudes. Participants first completed an environmental contingency measure and were randomly assigned to a death or control prime before completing the dependent measures of the study. Finally, participants completed some basic demographic information and probed for suspicion about the study. The materials in the study are presented in the order that participants completed them.

#### Environmental Contingent Self-Worth

ECSW was measured using 10 items (Brook, 2005) that examined the extent to which people saw their self-esteem as contingent on environmental outcomes (α = .92; e.g., “My self-esteem is influenced by how good or bad an environmentalist I am.”). Participants completed this on a seven-point scale (1 = *strongly disagree*; 7 = *strongly agree*).

#### Death Priming

To manipulate death thoughts, we utilized a manipulation used in Lifshin et al. (2017, Study 3) that consisted of participants rating three T-shirt designs. Participants in the death prime condition had the second T-shirt as an image of a skull made up of the word “death” printed multiple times. Participants in the control condition saw a different T-shirt that did not include any death themes.

#### Delay/Distraction

Participants completed the 20-item Positive and Negative Affect Schedule^3^ (PANAS; Watson et al., 1988) as a filler task before the dependent measures.

#### Human Superiority

We measured beliefs in human superiority over animals (Dhont & Hodson, 2014) with six items (α = .89; e.g., “*Animals are inferior to humans*”). Participants completed this measure on a nine-point scale (1 = *strongly disagree*; 9 = *strongly agree*).

#### Support for Animal Protection

We measured support for animal protection in six items (α = .91; e.g., “*It is duty of humans to protect animals*”) on a nine-point scale (1 = *strongly disagree*; 9 = *strongly agree*).

#### Support for Killing Animals

We measured support for killing animals with a highly face valid scale used in Lifshin et al. (2017, Study 1 and 2) that examines participants’ general support toward killing animals as well as in specific domains (e.g., research, population control, clothing). We used an abridged 11-item version of this scale (α = .89; e.g., “*An experiment should never cause the killing of animals*” (reversed item)). Responses were provided on a nine-point scale (1 = *strongly disagree*; 9 = *strongly agree*).

### Study 1 Results

We utilized Model 1 in PROCESS (A. F. Hayes, 2018) to test for the potential interactive effects of our death prime (−1 control, +1 death) and ECSW (mean centered) on support for killing and protecting animals as well as beliefs about human superiority. In line with Lifshin et al. (2017), we controlled for gender (dummy coded) in our models as it was a significant covariate.^4^

#### Animal Protection

**Figure 1.**
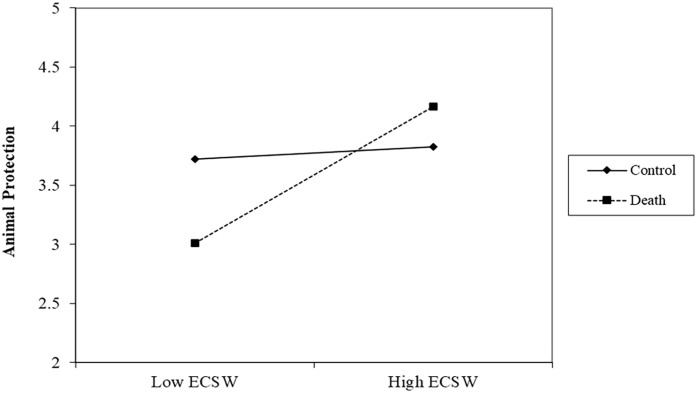
ECSW × Death Prime Interaction on Support for Animal Protection in Study 1. *Note.* ECSW = environmental contingent self-worth.

There was no effect of the death prime on support for protecting animals, *b* = −.09, *t* (192) = .86, *p* = .391, 95% confidence interval (CI): [−.30, .12]. There was a relationship of ECSW increasing support for protecting animals, *b* = .32, *t* (192) = 3.16, *p* = .002, 95% CI [.12, .51], which was qualified by the interaction, *b* = .26, *t* (192) = 2.67, *p* = .008, 95% CI [.07, .46] (See Figure 1). Simple slopes analyses showed that at low (-1 *SD*) levels of ECSW, the death prime decreased support for protecting animals, *b* = −.40, *t* (192) = 2.47, *p* = .015, 95% CI [−.71, −.08]. At high levels (+1 *SD*) of ECSW, the death prime increased support for protecting animals albeit this effect did not reach statistical significance, *b* = .21, *t* (192) = 1.39, *p* = .166, 95% CI [−.09, .51].

#### Killing Animals

**Figure 2.**
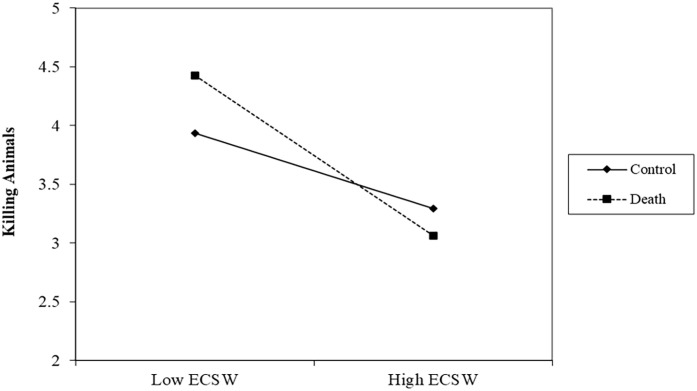
ECSW × Death Prime Interaction on Support for Killing Animals in Study 1. *Note.* ECSW = environmental contingent self-worth.

There was no effect of the death prime on support for killing animals, *b* = .07, *t* (192) = .59, *p* = .557, 95% CI [−.15, .28]. There was a relationship of ECSW decreasing support for killing animals, *b* = −.50 *t* (192) = 4.87, *p* < .001, 95% CI [−.70, −.30], which was qualified by a marginal interaction, *b* = −.18, *t* (192) = 1.80, *p* = .074, 95% CI [−.38, .02] (See Figure 2). At low levels of ECSW, the death prime marginally increased support for killing animals, *b* = .28, *t* (192) = 1.67, *p* = .096, 95% CI [−.05, .60]. At high levels of ECSW, the death prime decreased support for killing animals albeit not significantly, *b* = −.15, *t* (192) = .93, *p* = .353, 95% CI [−.46, .16].

#### Human-Animal Superiority

We performed the same analysis concerning our human-animal superiority measure. There was no effect of the death prime, *b* = −.11, *t* (192) = .82, *p* = .414, 95% CI [−.36, .15]. There was a relationship of ECSW decreasing beliefs about human superiority, *b* = −.69, *t* (192) = 5.71, *p* < .001, 95% CI [−.93, −.45]. The interaction effect was not significant, *b* = −.09, *t* (192) = .78, *p* = .434, 95% CI [−.33, .14].

### Study 1 Discussion

In comparison to Lifshin et al. (2017), we did not find that a death prime generally increased support to kill animals. Instead, the data were generally more consistent with our prediction. The death prime increased support for killing, and decreased support to protect, animals at low levels of ECSW. At high levels of ECSW, the reverse was true, although it should be noted that neither the simple effect nor the interaction reached conventional thresholds of statistical significance. One interpretation of these findings might be that high ECSW merely prevents the effects of death anxiety on increasing hostility toward animals rather than necessarily increasing a desire to protect and nurture them. While we cannot rule out this possibility based on the current data, this does not necessarily undermine our assertion that attitudes toward animals depend on what people see as relevant to their self-worth.

We found no support for our predictions that death primes would affect beliefs about human superiority over animals, even when considering ECSW as a potential moderator. This is inconsistent with some TM theorizing and research (e.g., Goldenberg et al., 2001) though could be considered consistent with findings from a recent replication (Rodríguez-Ferreiro et al., 2019). We sought to examine this issue further in Study 2.

## Study 2

As some of the findings of Study 1 were marginal, to increase confidence in our findings, we sought to replicate them in a new, larger sample. In addition, we also measured ATAR in Study 2, which was an important covariate in Lifshin et al’s (2017) work. It is possible that a clearer pattern of effects would emerge when controlling for this influence. Moreover, measuring ATAR allowed us to examine the specificity of our moderating effect to ECSW.

In addition, we sought to change our human superiority measure in Study 2. Although unlikely, one possibility for the findings might be that our measure did not appropriately capture a sense of superiority to animals. Another possibility might be because we measured human superiority rather than human–animal similarity. While literature in TMT tends to treat the two concomitantly (e.g., Goldenberg et al., 2000), it is possible that previous findings have been driven by a desire to feel different from animals, rather than necessarily superior to them. Thus, in Study 2, we also included a measure of human–animal similarity. Finally, because measuring human–animal similarities might cause downstream effects on our killing and protection measures, participants either completed our human–animal measures first or last.

### Study 2 Method

#### Participants

We recruited participants via social media and online forums (e.g., Facebook; reddit subforums relating to our nationality targets). No compensation was provided. We aimed to collect as much data as possible by a specified end date. To ensure a diverse sample, we constructed four identical surveys in English (*n* = 186), Hungarian (*n* = 206), Romanian (*n* = 53), and Spanish (*n* = 29).^5^ Analyses per country of origin are provided in the Supplementary Analyses. In total, 474 participants initiated the study, but only 397 completed it. We excluded 58 participants for the same criteria as Study 1, and a further 26 participants who indicated they had seen the manipulation before or indicated no T-shirt pictures appeared for them during the study. Therefore, the final sample consisted of 313 participants (*M*_age_ = 30.4, *SD*_age_ = 12.3) with 186 females, 119 males, and 7 identified as non-binary (1 participant did not provide this information). Post hoc sensitivity analyses suggested we had 90% power to detect a small-medium interaction effect f^2^ = .03. Participants were allocated to one of four conditions (control/human-animal first: *n* = 114; death/human-animal first: *n* = 121; control/human–animal last: *n* = 47; death/human–animal last: *n* = 31). Ethical approval was provided by the university.

#### Materials and Procedure

The study was almost identical to Study 1, with the only notable change being that we measured human–animal similarity alongside the human superiority measure. We also counterbalanced the order in which participants completed these measures so that participants either completed the human-animal measures first or last. Otherwise, participants completed the following materials in order.

##### Environmental Contingent Self-Worth

We measured ECSW using the same items from Study 1 (α = .93).

##### Death Priming

We manipulated death thoughts using the same T-shirt death prime (vs. control) that was used in Study 1.

##### Delay/Distraction

Participants completed the short version of the morningness–eveningness questionnaire (Smith et al., 1989) as a filler task.

##### Human-Animal Superiority and Similarity

We measured human superiority and similarity from measures similar to past research (Amiot et al., 2017). Three items assessed the extent to which people viewed humans as superior to animals (e.g., *“humans have a higher status compared to other animals”*) and three items assessed the extent to which people viewed humans as similar to animals (e.g., *“humans and animals are similar to each other”*). Participants responded to these items on a nine-point scale (1 = *strongly disagree*, 9 = *strongly agree*). We computed mean scores for superiority (α = .89) and similarity (α = .75).

##### Support for Animal Protection

We measured support for protecting animals with the same items used in Study 1 (α = .93).

##### Support for Killing Animals

We utilized the full 15 items from Lifshin et al. (2017) to measure support for killing animals (α = .90).

##### Attitudes Toward Animal Rights

As part of the demographic information at the end of the study, we captured ATAR using the same item as Lifshin et al. (2017).

### Study 2 Results

A preliminary analysis suggested that order^6^ did not qualify for any of our effects. Therefore, we collapsed by order and examined the death prime (−1 Control, +1 Death) × ECSW (mean centered) interaction using Model 1 in PROCESS (A. F. Hayes, 2018). In line with Lifshin et al. (2017), we controlled for gender (dummy coded) and ATAR^7^ which were significant covariates in our analyses.^8^

#### Animal Protection

**Figure 3.**
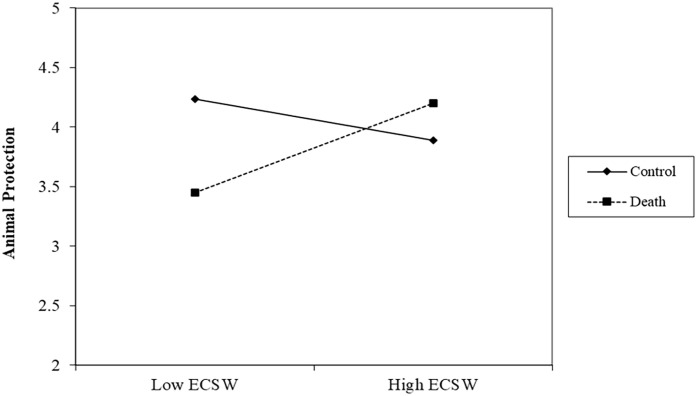
ECSW × Death Prime Interaction on Support for Animal Protection in Study 2. *Note.* ECSW = environmental contingent self-worth.

There was no effect of the death prime, *b* = −.12, *t* (305) = 1.49, *p* = .138, 95% CI [−.27, .04], or relationship of ECSW on protecting animals, *b* = .10, *t* (305) = 1.41, *p* = .158, 95% CI [−.04, .24]. Replicating Study 1, the interaction was significant, *b* = .27, *t* (305) = 4.22, *p* < .001, 95% CI [.15, .40] (See Figure 3). At low levels of ECSW, the death prime decreased support for protecting animals, *b* = −.45, *t* (305) = 4.04, *p* < .001, 95% CI [−.67, −.23]. At high levels of ECSW, the death prime marginally increased support for protecting animals, *b* = .22, *t* (305) = 1.94, *p* = .053, 95% CI [−.00, .44].

#### Killing Animals

**Figure 4.**
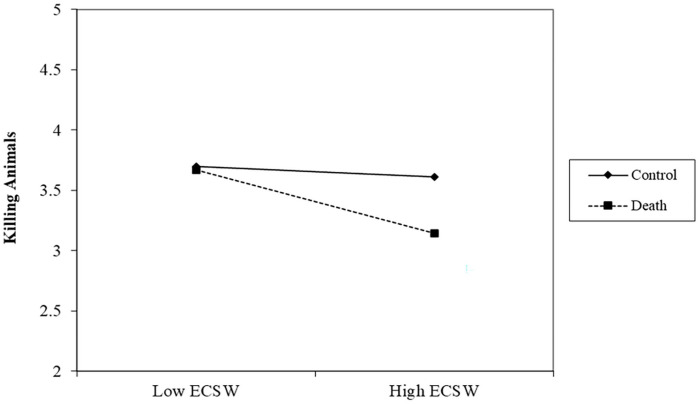
ECSW × Death Prime Interaction on Support for Killing Animals in Study 2. *Note.* ECSW = environmental contingent self-worth.

There was a significant effect of the death prime decreasing support for killing animals, *b* = −.15, *t* (305) = 2.02, *p* = .044, 95% CI [−.31, −.00]. There was a marginal relationship of ECSW decreasing support for killing animals, *b* = −.12, *t* (305) = 1.81, *p* = .072, 95% CI [−.26, .01]. The interaction was approaching significance, *b* = −.11, *t* (305) = 1.76, *p* = .080, 95% CI [−.23, .01] (See Figure 4). Examining this interaction showed that at low levels of ECSW, there was no effect of the death prime on support for killing animals, *b* = −.02, *t* (305) = .18, *p* = .854, 95% CI [−.23, .19]. At high levels of ECSW, the death prime significantly decreased support for killing animals, *b* = −.29, *t* (305) = 2.70, *p* = .008, 95% CI [−.50, −.08].

#### Human–Animal Superiority and Similarity

There was a marginal, albeit nonsignificant, effect of the death prime decreasing human superiority beliefs, *b* = −.24, *t* (305) = 1.84, *p* = .067, 95% CI [−.50, .02]. ECSW did not predict beliefs about human superiority, *b* = −.12, *t* (305) = 1.03, *p* = .306, 95% CI [−.35, .11]. The interaction was also not significant, *b* = −.12, *t* (305) = 1.15, *p* = .251, 95% CI [−.33, .09].

There was no significant effect of the death prime, *b* = .01, *t* (305) = .07, *p* = .943, 95% CI [-.20, .22], or relationship of ECSW, *b* = .00, *t* (305) = .05, *p* = .964, 95% CI [−.18, .19] on human–animal similarity. The interaction effect was also not significant, *b* = −.12, *t* (305) = 1.34, *p* = .181, 95% CI [−.28, .05].

#### Ancillary Analyses

ATAR and ECSW were only moderately correlated, *r* = .40, *p* < .001, providing discriminant validity to support that we measured different constructs. We examined whether our effects on support for killing and protecting animals could be explained by ATAR by performing the same analyses except switching out ECSW for ATAR as a moderating variable. None of the interactions were significant, *p*’s > .10, and the simple effects did not produce similar patterns to those observed when using ECSW as a moderator.

### Study 2 Discussion

Study 2 generally replicated our findings of Study 1. However, unlike Study 1, we found a main effect of the death prime *decreasing* support for killing animals. However, because this was qualified by an interaction between the death prime and ECSW, this is largely inconsequential. Interestingly, we found no support that those with low ECSW would show increased support for killing animals after a death reminder. These findings are both inconsistent with our hypothesis and Lifshin et al. (2017).

The findings also generally confirmed our findings of Study 1 that a death prime did not increase a sense of human superiority, and if anything, we observed a decrease. However, due to the marginal nature of this finding, we would caution over interpreting it. We also did not find any support that death primes affect beliefs regarding human-animal similarity. This is again inconsistent with past TMT research. We subsequently dropped these measures from our next study but discuss the implications of these findings in the general discussion.

## Study 3

We have so far found only marginal support that death primes increase support for killing animals. In Study 1, this tendency was limited to those low in ECSW and was marginal at best. In Study 2, we found no support for this effect. One major difference between our research and Lifshin et al. (2017) was the fact that we measured support for animal protection as well as support for killing. In both studies, all participants answered the protection measure first. We did so because measuring support for killing animals first could potentially prime death thoughts that produces a downstream effect on our protection measure (J. Hayes & Schimel, 2018). However, it is possible that answering the animal protection measure first increases the salience of such a norm which attenuates the support for killing animals. It might also be plausible that without measuring animal protection first, we might find that ECSW no longer moderates people’s support for killing animals. To discount this alternative interpretation, we randomized the order of the two measures in Study 3. We expected a clearer pattern of moderation effects to emerge on the measure that was answered first.

In addition, we also sought to extend our findings further by considering the downstream consequences of support for killing and protecting animals. Lifshin and colleagues (2017) found that death primes indirectly increased a sense of power-based invulnerability (PBI) through increased support for killing animals. We expected to replicate this effect although only for those with low ECSW. As with Lifshin et al. (2017), we did not anticipate direct effects of our death prime on PBI but expected that an indirect effect would statistically emerge.

Regarding high ECSW, we kept our prediction open. We suspected that if negative attitudes toward animals increases a sense of PBI, then increased prosociality toward animals would lead to a lower sense of PBI. However, we also envisaged an alternative possibility whereby the ability to nurture, care for, and watch over nature may elevate oneself above mere mortal status which could increase a sense of PBI.

### Study 3 Method

#### Participants

As our previous studies had small-medium effects, we powered to detect a small (f^2^ = .04) three-way interaction effect (1 focal predictor, 7 total predictors) at 90% power. This suggested we needed at least 265 participants. However, to ensure sufficient power after exclusions we recruited 298 American participants via Amazon MTurk. We excluded 38 participants who failed attention checks, and 5 participants who said we should not use their data. Therefore, the final sample consisted of 255 participants (*M*_age_ = 39.5, *SD*_age_ = 12.2) with 134 females and 121 males. Participants were allocated to one of our four conditions (control/protect first: *n* = 60; death/protect first: *n* = 63; control/kill first: *n* = 69; death/kill first: *n* = 63). Ethical approval was provided by the university.

#### Materials and Procedure

The study was pitched as an examination of personality and social attitudes. The procedure was similar to the previous studies aside from the following notable changes. First, we used a different death priming procedure. Second, we dropped our measures pertaining to human–animal similarity or superiority. Third, the order in which participants completed the measures regarding support for protecting and killing animals was counterbalanced. Fourth, at the end of the study we measured a sense of power- and control-based invulnerability.

##### Environmental Contingent Self-Worth

Participants first completed the measure of ECSW from the previous studies (α = .91).

##### Death Priming

To manipulate death thoughts, participants completed a 28-item word fragment task (J. Hayes & Schimel, 2018). In the experimental group, eight of these words could be completed in a death-related manner (dead, corpse, skull, grave, mortal, killed, buried, fatal).^9^ Participants in the control condition completed the same word fragment task except that the potential death words were switched out for additional neutral filler words.

##### Delay/Distraction

J. Hayes and Schimel (2018) demonstrated that word fragment tasks produce distal defenses after long (i.e., 2 tasks) delays. As we recruited participants from MTurk, who are known to provide speedy responses, we utilized three delay tasks. Participants completed the PANAS (Watson et al., 1988), morningness–eveningness questionnaire (Smith et al., 1989), and the short form Big Five personality inventory (Donnellan et al., 2006) to ensure thoughts of death had left focal attention.

##### Support for Protecting Animals

Participants completed the same measure from the previous studies (α = .92).

##### Support for Killing Animals

Participants completed the same measure from Study 2 (α = .91).

##### Power- and Control-Based Invulnerability

We utilized the five items from Lifshin et al. (2017) but added an additional item so that we measured power-based invulnerability (PBI; e.g., “I can escape dangerous situations”) and control-based invulnerability (CBI; e.g., “I have a great deal of control over my life”) with three items each. Responses were provided on nine-point scales (1 = *strongly disagree*, 9 = *strongly agree*). A principal components analysis supported these as separate factors, so we computed mean scores of PBI (α = .71) and CBI (α = .77).

### Study 3 Results

We utilized Model 3 in PROCESS (A. F. Hayes, 2018) to test for the interactive effect of the death prime and ECSW on support for killing and protecting animals. Prime (−1 Control, +1 Death), Order (−1 Protect first, +1 Kill first), and ECSW were mean centered in the model. As with the previous studies, we controlled for gender and ATAR which were significant covariates in our model.^10^

#### Animal Protection

**Figure 5.**
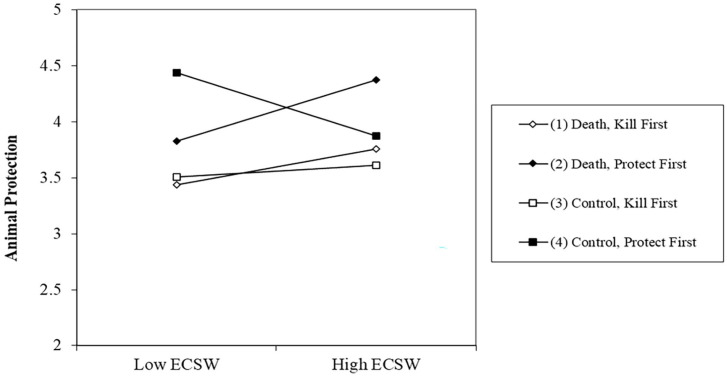
Effect of the Death Prime on Support for Animal Protection at High (+1 SD) and Low (−1 SD) ECSW as a Function of Dependent Variable Order. *Note.* SD = standard deviation; ECSW = environmental contingent self-worth.

There was no effect of the death prime on support for protecting animals, *b* = −.01, *t* (244) = .06, *p* = .952, 95% CI [−.17, .16]. There was an effect of order whereby completing the kill measure first decreased willingness to protect animals, *b* = −.27, *t* (244) = 3.33, *p* = .001, 95% CI [−.43, −.11]. The relationship of ECSW was not significant, *b* = .05, *t* (244) = .66, *p* = .509, 95% CI [−.10, .20]. The Death Prime X ECSW interaction was significant, *b* = .17, *t* (244) = 2.51, *p* = .013, 95% CI [.04, .30], which was marginally qualified by a Death Prime × Order × ECSW interaction, *b* = −.11, *t* (244) = 1.71, *p* = .089, 95% CI [−.24, .02] (see Figure 5).

Examining the simple effects of this three-way interaction showed that when protection was measured first, the death prime decreased the desire to protect animals for those with low ECSW, *b* = −.38, *t* (244) = 2.34, *p* = .020, 95% CI [−.69, −.06]. The death prime marginally increased the desire to protect animals for those high in ECSW when it was measured first, *b* = .32, *t* (244) = 1.91, *p* = .057, 95% CI [−.01, .65]. There were no effects when the kill measure was answered first, *p*’s >.50.

#### Killing Animals

There was no effect of the death prime, *b* = −.07, *t* (244) = .84, *p* = .402, 95% CI [−.24, .09], or Order, *b* = −.09, *t* (244) = 1.07, *p* = .288, 95% CI [−.25, .08] on support for killing animals. The relationship of ECSW decreasing support for killing animals was marginal, *b* = −.14, *t* (244) = 1.75, *p* = .082, 95% CI [−.28, .02]. The two-way interaction between Death Prime × Order was not significant, *b* = .14, *t* (244) = 1.64, *p* = .103, 95% CI [−.03, .30]. The Death Prime × ECSW interaction was significant, *b* = −.22, *t* (244) = 3.25, *p* = .001, 95% CI [−.35, −.09]. The three-way interaction however was not significant, *b* = −.09, *t* (244) = 1.31, *p* = .191, 95% CI [−.22, .04].

**Figure 6.**
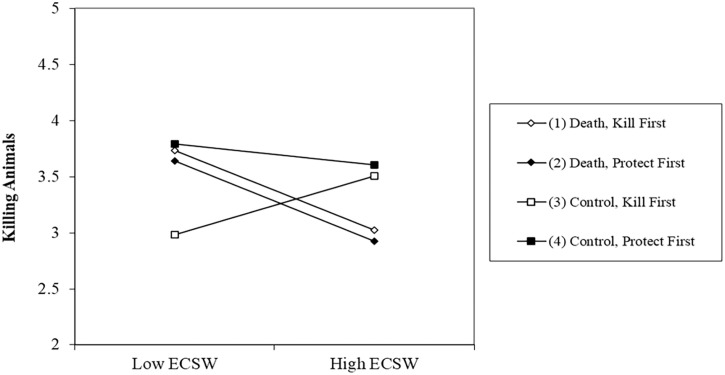
Effect of the Death Prime on Support for Killing Animals at High (+1 SD) and Low (−1 SD) ECSW as a Function of Dependent Variable Order. *Note.* SD = standard deviation; ECSW = environmental contingent self-worth.

Noting the nonsignificant three-way interaction, we continued with our planned analysis strategy to examine the simple effects (see Figure 6). These did suggest that our order manipulation meaningfully affected the Death Prime X ECSW interaction albeit only for those low in ECSW. When kill was measured first, the death prime increased support for killing animals among those with low ECSW, *b* = .45, *t* (244) = 2.61, *p* = .010, 95% CI [.11, .79]. This effect was not significant when protection was measured first, *b* = −.04, *t* (244) = .27, *p* = .788, 95% CI [−.37, .28]. In comparison, the order of the measures did not influence those high in ECSW. Participants high in ECSW displayed a decreased willingness to kill animals after the death prime when measured first, *b* = −.32, *t* (244) = 1.92, *p* = .056, 95% CI [−.64, .01], and when measured last, *b* = −.37, *t* (244) = 2.18, *p* = .030, 95% CI [−.71, −.04].

#### Power- and Control-Based Invulnerability

Despite not having predictions regarding the direct effects of our death prime on PBI/CBI, examining the direct effects suggested that there was a significant Death Prime × Order interaction on PBI, *b* = .22, *t* (244) = 2.05, *p* = .041, 95% CI [.01, .42]. The Death Prime × Order × ECSW interaction was not significant, *b* = −.14, *t* (244) = 1.64, *p* = .102, 95% CI [−30 .03]; however, the simple slopes were interpretable. These showed that the death prime increased a sense of PBI for those with low ECSW, but only when kill was measured first, *b* = .50, *t* (244) = 2.31, *p* = .022, 95% CI [.07, .93]. All other slopes were not significant, *p*s > .15. There were no direct effects when examining CBI.

To assess whether the death prime had an indirect effect on levels of PBI and CBI depending on levels of ECSW, we used Model 12 in PROCESS (A. F. Hayes, 2018). This tests for a three-way interaction on PBI/CBI through our mediating variables (protect/kill). As with the main analysis we included gender and ATAR as covariates. To examine any indirect effect, we utilized bootstrapping analyses using 10,000 samples.^11^

The indirect effect (IE) of the death prime on PBI through protection was significantly different from zero, but only when protection was measured first. More specifically, the death prime indirectly increased a sense of PBI for those with low ECSW, IE = .10, 95% CI [.01, .22]. The death prime indirectly decreased a sense of PBI for those with high ECSW, IE = .09, 95% CI [−.19, −.00]. There was no indirect effect of the death prime through protection when the kill was measured first.

We observed a similar pattern of effects concerning the kill variable as a mediator. The indirect effect of the death prime on PBI was mediated by support for killing, particularly when support for killing was measured first. The death prime indirectly increased a sense of PBI for those with low ECSW when the kill was measured first, IE = .10, 95% CI [.00, .24]. The indirect of the death prime decreased a sense of PBI for those with high ECSW when kill was measured first, IE = −.07, 95% CI [−.19, .01], although this effect was only significant at a 90% CI level. Finally, the death prime also indirectly decreased a sense of PBI for those with high ECSW when protection was measured first, IE = −.08, 95% CI [−.18, −.01]. We observed no effects on the CBI variable.

#### Ancillary Analyses

ATAR and ECSW were moderately correlated, *r* = .49, *p* < .001. ATAR did not moderate our effects on support for killing animals, *p*’s > .50.

However, there was a marginal Death Prime X ATAR interaction on support for protecting animals, *b* = .60, *t* (245) = 1.84, *p* = .067, 95% CI [-.04, 1.25]. The three-way interaction was not significant, *p* >.15 but showed a similar, albeit weaker pattern to the effects reported in the main analysis. To further disentangle the effects of ATAR and ECSW, we ran a hierarchical regression model that included the interaction effects for both variables. The interaction effect for ATAR was now highly nonsignificant, while the general pattern of the effects for ECSW remained the same.

### Study 3 Discussion

The results of Study 3 were generally consistent with our previous findings and predictions. Notably, we found much stronger support for the death prime increasing support for killing animals when this was measured first, but consistent with our prediction this was limited to those with low ECSW. In comparison, there was no evidence that the death prime increased support for killing animals when protection was measured first. This helps clarify our previous findings by suggesting that the protection measure might have heightened environmental-based norms that decreased support for killing animals, particularly for those with low ECSW. Presumably, these environmental-based norms would be chronically accessible for those with environmental contingencies hence why little evidence of an order effect was found in these participants.

Our findings of Study 3 also suggested that death primes indirectly influence a sense of PBI through support for killing and protecting animals. This is consistent with Lifshin et al. (2017). However, we found that this increase in PBI was only the case for those with low ECSW. In contrast, those with high ECSW showed a lower sense of PBI through their decreased support for killing, and increased support for protecting, animals. Thus, it seems that increased support for animals might come with a psychological consequence of feeling more vulnerable and less powerful.

## Combined Data Analysis

To provide further corroborating support for our hypotheses, we conducted a pooled data analysis of Studies 1 to 3. We standardized all variables and then combined them into one dataset (*n* = 765). Because we did not include ATAR in Study 1, we only analyzed the data controlling for gender which we had data for in all samples. In addition, as the order of the kill and protection measures seemed to meaningfully influence the results in Study 3, we only included the data when that dependent variable was measured first.

**Figure 7.**
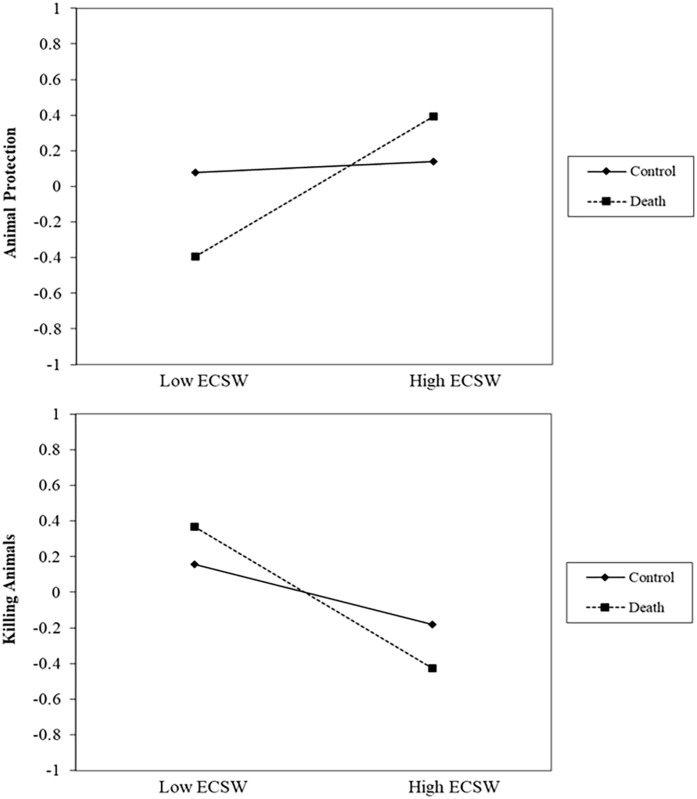
Findings of the Combined Analysis Regarding the Death Prime × ECSW Interaction Effects on Support for Protecting and Killing Animals. *Note.* ECSW = environmental contingent self-worth.

Regarding support for animal protection, the combined analysis yielded a significant death prime × ECSW interaction effect, *b* = .18, *t* (627) = 5.01, *p* < .001, 95% CI [.11, .25] (see Figure 7). At low levels of ECSW, the death prime decreases support for protecting animals, *b* = −.24, *t* (627) = 4.64, *p* < .001, 95% CI [−.34, −.14]. At high levels of ECSW, the death prime increases support for protecting animals, *b* = .13, *t* (627) = 2.47, *p* = .014, 95% CI [.03, .23].

The combined analysis also produced a significant death prime × ECSW interaction effect on support for killing animals, *b* = −.11, *t* (641) = 3.15, *p* = .002, 95% CI [−.19, −.04] (see Figure 7). At low levels of ECSW, the death prime increase support for killing animals, *b* = .10, *t* (641) = 2.02, *p* = .044, 95% CI [>.00, .20]. At high levels of ECSW, the death prime decreases support for killing animals, *b* = −.12, *t* (641) = 2.46, *p* = .014, 95% CI [−.22, −.02].

Thus, the combined analysis provides robust support for our hypothesis that people’s support to protect, or kill, animals after being reminded of death depends on whether their self-worth is contingent on environmental issues. A pooled analysis using ATAR as a moderator did not produce any significant interactions or simple effects.

Regarding human superiority, the overall interaction term was not significant, *b* = −.06, *t* (504) = 1.53, *p* = .128, 95% CI [−.15, .02]. but the analysis suggests that the death prime significantly reduces beliefs about human superiority among those with high ECSW, *b* = −.13, *t* (504) = 2.27, *p* = .024, 95% CI [−.25, −.02]. While we would caution not overinterpreting this finding, this effect could be consistent with our other findings as it might suggest that people’s willingness to be prosocial toward animals is because they do not see themselves as superior to them.

## General Discussion

Lifshin and colleagues (2017) proposed that support for killing animals may serve a terror management function as it can help people feel superior to animals and deny their creatureliness. Our findings are partially consistent with this claim by demonstrating that, under specific conditions, death primes may cause decreased support toward animals. However, our findings also provide a more nuanced picture to the role of death primes in animal-related attitudes.

First, three studies consistently favored our hypothesis regarding how the effect of death primes on animal-based attitudes depends on one’s level of ECSW. In contrast, we failed to identify the main effect of death primes increasing support for killing animals. Despite this, we emphasize that we do not believe our research should be considered a failed replication of Lifshin et al. (2017). Indeed, if anything, our interactional prediction would probably suggest that a main effect might be detectable in some samples but less detectable in others. Perhaps, the participants in Lifshin et al. (2017) studies generally had lower ECSW. More generally, environmental concerns and attitudes have increased globally in a short space of time. For example, a nationally representative survey of Europeans over the last decade shows an increasing number of people see environmental issues as a “very serious concern” (78% vs. 68%; European Commission, 2021). This is matched by data in the United States where the number of people reporting they are worried a “great deal” about the environment over the last decade has risen (43% vs. 30%; Gallup, 2022). Similarly, veganism and other pro-animal attitudes have increased during this time too (Wunsch, 2021). Perhaps these changes reflect cultural shifts that lead to less support for killing animals (see Ruby, 2012) and corresponds with research demonstrating that norms legitimizing animal harm are important in shaping human-animal relations (e.g., Bègue & Vezirian, 2021). In the context of our research, this might suggest that there is a trend toward self-worth becoming increasingly contingent on environmental issues. Indeed, our sample average hovered above the theoretical midpoint of the ECSW scale suggesting the typical participant’s self-worth was somewhat contingent on environmental issues, whereas past research shows that participants report below the theoretical midpoint (Brook, 2011). Perhaps this shift might have been significant enough to have made a main effect of death primes harder to detect in our studies.

Another possibility pertains to a key methodological difference in our study and Lifshin and colleagues’ (2017). First, only one experiment by Lifshin et al. (2017, Study 3) was conducted online using the T-shirt manipulation, and that study deployed a different dependent measure to the killing animals scale. Their samples were also all college students, while our samples were recruited online. It is possible therefore that with different design choices, we might have found more support for their original effect. Second, and most notably, participants always completed the ECSW at the beginning, while Lifshin and colleagues (2017) measured ATAR in a pre-screening survey several weeks before the study. We would note that despite this, in Studies 2 and 3 when we did measure ATAR, we found little support for its moderating effect. However, one possibility is that measuring ECSW within the main study might have increased the salience of environmental-based issues that dampened the effects on the support to kill animals. This is supported by a recent meta-analysis of salient norms shaping reactions to mortality salience (Schindler et al., 2022). Indeed, the findings of Study 3 might add further credence to this possibility, as measuring animal protection first attenuated the support for killing animals, so perhaps the environmental contingency measure might have produced a similar effect. However, while this may have dampened our effects, it should be noted that we generally managed to identify the effects of decreased pro-animal relations among those with low ECSW. Nonetheless, future work that examines the role of ECSW should consider measuring it in the prescreening survey or at the end of the study if the moderator is assumed to be unaffected by the manipulation.

While this issue might explain the weaker support for Lifshin et al.’s (2017) finding, we believe it does not undermine the central assertion of our research which is that reactions to animals depend on the contingencies promoted within one’s worldview and that death primes can therefore produce increased support for protecting nonhuman animals. While measuring ECSW at the beginning of the study may have activated environmental norms that dampened effects in low ECSW, it likely had little effect on those with high ECSW as important contingencies of self-worth are more chronically accessible to the individual (Crocker & Wolfe, 2001; Higgins, 1996) and thus are less reliant on situational cues. Study 3 illustrates this point as the order of the two dependent measures did not affect the extent to which participants high in ECSW were likely to support the killing of animals.

Of course, future work should seek to examine the role of salient environmental norms in human-animal relations as this went beyond the purview of our investigation, which was to focus on dispositional differences in ECSW. Nonetheless, our findings do suggest that norm salience may be important. Moreover, while salient norms may temporarily heighten support for animals, this may be manifested into a longer term commitment when such norms become the basis for one’s self-worth. These findings, therefore, suggest that promoting pro-animal values and policies may not only decrease the effect of death anxiety on increasing aggression toward animals but may even reverse its effect. This finding may be of importance to policymakers and public officials who hope to shift public perception of animals and animal welfare (Lifshin, 2022; Marino & Mountain, 2015).

Our findings demonstrated that animal protection is linked with decreased feelings of personal power and invulnerability. Thus, our findings suggest there may be a trade-off between social (e.g., aggression toward animals) and personal (e.g., psychological security) outcomes when managing death anxiety. One potential implication of this finding is that while it is possible for worldviews to espouse positive attitudes toward animals, it may come at the price of feeling less personal existential security. This complements past research which suggests that people with low perceived similarity to animals tend to be more nationalistic and prejudiced, while those with high perceived similarity to animals tend to experience greater anxiety (Lifshin et al., 2021). Future research should examine this potential trade-off further. For example, this trade-off may be limited to Western cultures where typically separating oneself from animals is necessary for death transcendence (Lifshin & Greenberg, 2019).

TMT suggests that people seek to feel superior and separate from nature, which can lead to a disidentification with one’s animal status (Goldenberg et al., 2000). We had originally conceived the sense of human superiority and human–animal similarity as possible mediators in our effects. However, our research did not support this conclusion and more generally did not support the idea that mortality salience increases a tendency to see humans as separate, and superior, to animals (Goldenberg et al., 2001). Of course, our study was very different from the Goldenberg et al. (2001) study. We did not use an explicit mortality salience prime, and our measurement of human–animal beliefs consisted of direct statements rather than using reactions to essays that emphasized human-animal similarity/distinctiveness, and our study was conducted online rather than in the laboratory. Nonetheless, our findings are potentially consistent with a recent replication of the Goldenberg et al. (2001) that also did not find evidence to support this effect (Rodríguez-Ferreiro et al., 2019).^12^

One interpretation of these findings is that mortality salience does not increase a tendency to deny one’s animal status and that past research that supported this effect was a false positive. However, we do not think that this is the case. First, measuring these constructs with direct statements might have elicited socially desirable responding that examining reactions to essays might alleviate. Second, our measures tapped into the extent to which one perceives humans in general to be similar to animals, but it may be more important to consider the extent to which one perceives their self in relation to animals (Lifshin et al., 2021). This latter explanation is compatible with the findings of the present study that show it may be more useful for terror management researchers to focus on personal measures of the self and self-worth rather than impersonal measures regarding worldview beliefs.^13^

Beyond these methodological reasons, we suspect that there may be several conditions, other than just mortality salience, that may produce a tendency to disidentify with animals and/or assert a sense of superiority over them. Indeed, reactions to mortality salience are contingent on several factors including values and beliefs that are promoted in one’s cultural worldview. For example, a Gallup poll suggests a third of Americans believe that animals should have equal rights to humans (Jones & Saad, 2015). Thus, mortality salience effects are never really main effects (Pyszczynski et al., 2015). Yet research examining TMT and human-animal beliefs have generally not yet examined moderators to the tendency to deny one’s creatureliness. While our research does not provide robust support for the idea that ECSW moderates the effect of death primes on these constructs, our pooled analysis does provide initial evidence that perhaps certain worldviews may decrease beliefs regarding human-animal superiority after death primes. Perhaps it would be fruitful for researchers to investigate other sorts of beliefs or personality styles that might moderate these effects. For example, a sense of empathy (Hills, 1995), the tendency to anthropomorphize animals (Butterfield et al., 2012), and social dominance orientation (Costello & Hodson, 2010) might be interesting moderators to examine as they are associated with beliefs regarding human–animal similarity and/or superiority. Similarly, trait differences in the tendency to perceive oneself as similar to animals may also moderate the extent to which people deny their animal status after death primes (Lifshin et al., 2021).

Our research does have some limitations. Perhaps most importantly, we are unable to provide causal evidence for the moderating role of ECSW. Similarly, the results of Study 3 cannot provide definitive causal evidence for how the support for killing and protecting animals can change feelings regarding perceived invulnerability. While manipulating contingencies of self-worth would be ideal, unfortunately, this would be impractical to achieve due to how relatively stable contingencies of self-worth are over time (e.g., Crocker et al., 2003). Another limitation concerns the use of subtle death primes, which were also used by Lifshin et al. (2017). We opted to do this to reduce the possibility of participants connecting the death prime to the killing animals measure. However, it is unclear therefore whether such findings would hold when using more explicit, conscious death primes that are more traditionally deployed in TMT research. A final limitation of this research concerns the measurement of ATAR in one item. It might be possible that our ECSW measure was a more sensitive moderator in comparison to ATAR because we captured ECSW with several items. We cannot discount this possibility but would note that this issue does not alter the take-home message of this research which is that how people respond to death primes depends on an individual’s contingencies of self-worth that are rooted in one’s belief system.

The central implication of this research is that benevolent, as well as hostile, attitudes toward animals may serve a terror management function. When one’s self-worth is pitted on environmental concerns, this can shape terror management defenses to be more positive toward animals. This knowledge may be fruitful in trying to increase support for animal and environmental-based issues. Charities and public officials may want to ensure messaging surrounding animal protection emphasizes the cultural value to be gained from such behaviors. From a social science perspective, understanding further how to promote environmental contingencies of self-worth may help to ensure such messaging is effective. By doing so, this may promote more support for protecting, rather than killing, animals.

## Supplemental Material

sj-docx-1-psp-10.1177_01461672231160652 – Supplemental material for To Protect or to Kill? Environmental Contingent Self-Worth Moderates Death Prime Effects on Animal-Based Attitudes Supplemental material, sj-docx-1-psp-10.1177_01461672231160652 for To Protect or to Kill? Environmental Contingent Self-Worth Moderates Death Prime Effects on Animal-Based Attitudes by Samuel Fairlamb, Andrada-Elena Stan and Katinka Lovas in Personality and Social Psychology Bulletin

## Notes

1. More specifically, they found that gender, attitudes toward killing animals and animal rights, perceived human–animal similarity, religiosity, political orientation, or the degree to which killing was justified did not moderate these effects (Lifshin et al., 2017). We focus specifically on attitudes toward animal rights in this study because it is conceptually the most similar to the environmental contingencies of self-worth measure that we examine here. Nonetheless, when we had data available for these other moderators, we reexamined their moderating effect and found no reliable results (*p*s > .05).

2. While our protection measure consistently yielded a reliable one factor solution, the killing animals measure load across several factors. However, as it formed a reliable composite measure, and we had no specific hypotheses regarding components of killing, and moreover, our primary interest was to compare our effects to Lifshin et al. (2017) who utilized it as an overall measure, we ignored these differences. In general, analyzing these components separately does not qualify the main conclusions from the report. We present the findings for these specific factors in the supplementary analyses for interested readers.

3. Analysis of affect in Studies 1 and 3 shows it was not affected by the death prime.

4. In the present studies, we controlled for gender and ATAR to be consistent with research conducted by Lifshin et al. (2017) and in personal correspondence was suggested to be important when analyzing the data (U. Lifshin, personal communication, March 25, 2021). In the interest of transparency, however, we briefly summarize the findings without controlling for these variables. Largely, the findings remain unaffected, but in some cases the findings weaken. Running a combined analysis across all datasets without controlling for gender or ATAR, shows that the Death Prime × ECSW interaction was significant for the protection variable, *b* = .19, *t* (629) = 5.03, *p* < .001, 95% CI [.11, .26] (Simple effects—low ECSW: *b* = −.24, *t* (629) = 4.58, *p* < .001 95% CI [−.35, −.14]; High ECSW: *b* = .13, *t* (629) = 2.53, *p* = .011, 95% CI [.03, .24], and the kill variable, *b* = −.12, *t* (642) = 3.16, *p* = .002, 95% CI [−.19, −.05] (Simple effects—low ECSW: *b* = .11, *t* (642) = 2.08, *p* = .038, 95% CI [.01, .22]; High ECSW: *b* = −.13, *t* (642) = 2.42, *p* = .016, 95% CI [−.23, −02].

5. Due to an error, we did not counterbalance the order of the human-animal measures in the Hungarian version so that participants in this survey always completed those measures first. However, as our findings did not suggest that the order of the measures meaningfully affected our results, we feel confident that this did not influence our findings.

6. There was a significant effect of the order of measures on killing animals, *b* = −.29, *t* (301) = 3.13, *p* = .002, 95% CI [−.47, −.11], with those completing the human-animal measures first showing increased support for killing animals. However, this did not interact with the death prime so does not qualify the findings reported.

7. Controlling for ATAR allows us to specify our effects are specific to ECSW. The same principle can be applied to using political orientation as a covariate which we collected in all studies. Controlling for political orientation does not alter the findings, and in some cases, marginally improves them with some reported effects reaching conventional significance (*p* < .05).

8. One participant who did not provide their gender and one participant who did not respond to the ATAR item were excluded from the analyses.

9. While we did not hold a specific prediction regarding whether differences in death-thought accessibility (DTA) would predict our dependent measures, one might suspect that our death prime was more effective when participants found more of the death words. Examining the death group provided marginal support for this view as there were significant DTA × ECSW interaction effects on our dependent variables. These showed that DTA negatively predicted protection for those with low ECSW, and positively predicted protection for those with high ECSW. The same pattern was found when examining the kill variables albeit the slopes were not significant (see Supplementary Analyses).

10. One participant did not answer the ATAR item and thus were excluded from the analyses.

11. Examining the reverse model (i.e., Death Prime → PBI → Protect/Kill) did not produce any reliable indirect effects that were significantly different from zero. This corresponds with Lifshin et al. (2017).

12. While Rodríguez-Ferreiro et al. (2019) failed to replicate Goldenberg et al. (2001), it is worthwhile noting that the replication did not include the threat essay regarding humans and animals being similar, and only examined reactions to the essay regarding humans and animals as different.

13. We would like to thank an anonymous reviewer for this suggestion.
