# Supplementary material for: To Protect or to Kill? Environmental Contingent Self-Worth Moderates Death Prime Effects on Animal-Based Attitudes
Source: Pers Soc Psychol Bull. 2023 Mar 21;50(9):1315–31. doi: 10.1177/01461672231160652 (PMC11318199; doi:10.1177/01461672231160652)
Supplement: sj-docx-1-psp-10.1177_01461672231160652 – Supplemental material for To Protect or to Kill? Environmental Contingent Self-Worth Moderates Death Prime Effects on Animal-Based Attitudes [file sj-docx-1-psp-10.1177_01461672231160652.docx]

**Studies 1-3: Factor Analysis**

As noted in the main manuscript (see footnote 2), we ran principal component analyses of the kill and protection measures in studies 1-3 using varimax rotation. For the protection measure, a one factor solution was extracted each time with all items loading strongly in that factor [Eigenvalues: 4.2 (Study 1), 4.7 (Study 2), 4.7 (Study 3)]. For the kill measure, several factors were extracted. For ease of interpretation, we only present the factor loadings that were > .50 (see Table SA.1). In Study 1 and 2, three factor solutions were extracted. In Study 3, a four-factor solution was extracted, but the fourth factor has a low eigenvalue (1.06) contributing to a small proportion of explained variance and the majority of items in this factor were loaded better the other three factors. Only two items loaded better in this factor (“animals should never be killed for their fur or leather” and “cosmetic research on animals that harms them is always unnecessary”). Thus, this factor related to killing of animals for materialistic consumption, but we disregarded this factor due to it only being comprised of two items and because it did not emerge in Study 2.

Aside from a few discrepancies (see Table SA.1), the items in each factor were consistent across studies and revolved around three thematic labels. The first factor concerned animal-based research (e.g., “An experiment should never cause the killing of animals”). The second factor concerned morals and traditions regarding human treatment of animals (e.g., “Killing animals is always wrong”). The third factor referred to culling animal population (e.g., “Because of pet overpopulation, animal shelters cannot help euthanizing unwanted pets”). We computed scales that reflected each factor^^[[1]](#footnote-1)^^, and analysed the data examining each kill factor separately.

**Table SA.1.** Factor loadings (only >.50 are displayed) for analyses of the kill measure in studies 1-3.

|  | **Study 1** | | | **Study 2** | | | **Study 3** | | |
| --- | --- | --- | --- | --- | --- | --- | --- | --- | --- |
|  | **F1** | **F2** | **F3** | **F1** | **F2** | **F3** | **F1** | **F2** | **F3** |
| Most animal research is wrong and should be stopped immediately | .88 |  |  | .85 |  |  | .69 |  |  |
| Since many important questions cannot be answered by doing experiments on people, we are left with no alternatives but to do animal research even if it has lethal consequences | .70 |  |  | .78 |  |  | .70 |  |  |
| An experiment should never cause the killing of animals | .74 |  |  | .76 |  |  | .61 |  |  |
| The fact that most animals die during or after an experiment does not mean that most of the experiments that are done on animals should be stopped* |  |  |  | .76 |  |  | .71 |  |  |
| Animal research is an important part of scientific progress and it should be continued | .66 |  |  | .60 |  |  |  |  |  |
| Cosmetic research on animals that harms them is always unnecessary* |  |  |  |  |  |  | .63 |  |  |
| It is human nature to kill other animals and thus the killing of other animals should not be seen as something so negative |  | .87 |  |  | .79 |  |  |  | .65 |
| Animals should never be killed for their fur or leather |  |  |  |  | .72 |  |  | .67 |  |
| People need to do much more than they are doing to prevent any kind of killing of animals* |  |  |  |  | .69 |  |  | .75 |  |
| Since the dawn of humanity, people have been killing animals for many purposes, and therefore trying to stop the killing of animals is impossible and absurd |  | .80 |  |  | .62 |  |  |  | .62 |
| Killing animals is always wrong |  |  | .53 |  | .58 |  |  | .80 |  |
| People should never kill animals because of overpopulation |  |  | .82 |  |  | .78 |  | .75 |  |
| It is often necessary to control for animal overpopulation through different means, such as hunting, or euthanasia |  |  | .75 |  |  | .77 |  |  | .80 |
| Overpopulated seal colonies should be sometimes narrowed down through hunting in order to make sure there are plenty of fish in the sea* |  |  |  |  |  | .74 |  |  | .84 |
| Because of pet overpopulation, animal shelters cannot help euthanizing unwanted pets |  |  | .70 |  |  | .63 |  |  | .78 |

*not measured in Study 1.

The analysis of each killing subscale in Studies 1-3 is presented in Table SA.2. As the main report suggested that the order of the measures in Study 2 did not meaningfully change the findings, for parsimony we dropped this variable from our analyses. In general, the pattern of the findings is consistent across the measures, though generally weaker than the composite measure utilised in the main analysis.

**Table SA.2:** Analysis of the kill variable by each subscale across studies 1-3. Numbers in the table reflect unstandardised coefficients.

|  | **Study 1** | | | **Study 2** | | | **Study 3** | | |
| --- | --- | --- | --- | --- | --- | --- | --- | --- | --- |
|  | **Animal Research** | **Morals and traditions** | **Animal population** | **Animal Research** | **Morals and traditions** | **Animal population** | **Animal Research** | **Morals and traditions** | **Animal population** |
| MS | .09 | .08 | -.05 | -.16 | -.12 | -.17 | -.11 | -.05 | -.08 |
| ECSW | -.54** | -.75*** | -.69*** | -.21^†^ | -.23* | -.10 | .04 | -.36** | -.21 |
| Order | - | - | - | - | - | - | -.02 | -.16^†^ | -.11 |
| MS X ECSW | -.22 | -.22 | -.27^†^ | -.15 | -.13 | -.19^†^ | -.23* | -.17^†^ | -45*** |
| MS X Order |  |  |  |  |  |  | .18^†^ | .16 | .12 |
| Order X ECSW |  |  |  |  |  |  | .25* | .11 | -.05 |
| MS X Order X ECSW |  |  |  |  |  |  | -.10 | -.09 | -.13 |
|  |  |  |  |  |  |  |  |  |  |
| *Simple Slopes (MS X ECSW)* |  |  |  |  |  |  |  |  |  |
| Low ECSW | .30 | .30 | .22 | -.01 | .01 | .02 | .12 | .12 | .35* |
| High ECSW | -.13 | -.14 | -.32 | -.30* | -.25^†^ | -.36* | -.33* | -.22 | -.52** |
|  |  |  |  |  |  |  |  |  |  |
| *Simple Slopes (MS X Order X ECSW)* |  |  |  |  |  |  |  |  |  |
| Protect First/Low ECSW | - | - | - | - | - | - | -.15 | -.13 | .11 |
| Protect First/High ECSW | - | - | - | - | - | - | -.42^†^ | -29 | -.52* |
| Kill First/Low ECSW | - | - | - | - | - | - | .41^†^ | .37^†^ | .62** |
| Kill First/High ECSW | - | - | - | - | - | - | -.27 | -.17 | -.54* |

****p* < .001; ** *p*< .01; * *p*< .05; ^†^*p* < .10

**Study 2: Country Analysis**

In study 2, we collected data from four different European countries and conducted a pooled analysis. We provide the effects per country as Supplementary data. Concerning our key measures, participants from different speaking countries did not differ on levels of ECSW. However, there were some effects on dependent variables concerning country of origin. These essentially suggested that the Romanian sample had higher levels of support for protection and lower support for killing animals than the other three combined, *p* ‘s <.05. Despite this, controlling for country (dummy coded 1 = Romania, 2 = other) in the primary analyses did not alter the findings presented in the main report.

We also present the primary analyses by each country (see Table SA.3), though would caution readers from interpreting the Spanish (n = 20) and Romanian (n = 33) samples which were unacceptably small. The English and Hungarian samples show reasonably consistent pattern of effects, with the effects stronger in the English sample. However, it is difficult to ascertain whether the strength of the effects is because of varying sample sizes or cultural differences.

**Table SA.3.** Analysis of the effects on animal protection and killing animals by individual country in Study 2. Numbers in the table are unstandardised coefficients.

|  | English | Hungarian | Romanian | Spanish |
| --- | --- | --- | --- | --- |
| Human-animal similarity | Prime X ECSW: *b* = .09  **Simple effects:**  Low ECSW: *b* = -.14  High ECSW: *b* = .05 | Prime X ECSW: *b* = -.06  **Simple effects:**  Low ECSW: *b* = .14  High ECSW: *b* = .02 | Prime X ECSW: *b* = -1.10**  **Simple effects:**  Low ECSW: *b* = 1.38*  High ECSW: *b* = -.80 | Prime X ECSW: *b* = -.66  **Simple effects:**  Low ECSW: *b* = .50  High ECSW: *b* = -.83 |
| Human-animal superiority | Prime X ECSW: *b* = -.32  **Simple effects:**  Low ECSW: *b* = .02  High ECSW: *b* = -.62* | Prime X ECSW: *b* = -.23  **Simple effects:**  Low ECSW: *b* = .07  High ECSW: *b* = -.40 | Prime X ECSW: *b* = .26  **Simple effects:**  Low ECSW: *b* = -1.03  High ECSW: *b* = -.52 | Prime X ECSW: *b* = .53  **Simple effects:**  Low ECSW: *b* = -.15  High ECSW: *b* = .92 |
| Protecting animals | Prime X ECSW: *b* = .59**  **Simple effects:**  Low ECSW: *b* = -.75***  High ECSW: *b* = .43* | Prime X ECSW: *b* = .16  **Simple effects:**  Low ECSW: *b* = -.19  High ECSW: *b* = .45 | Prime X ECSW: *b* = -.05  **Simple effects:**  Low ECSW: *b* = -.18  High ECSW: *b* = -.29 | Prime X ECSW: *b* = .36  **Simple effects:**  Low ECSW: *b* = -.51  High ECSW: *b* = .21 |
| Killing animals | Prime X ECSW: *b* = -.28*  **Simple effects:**  Low ECSW: *b* = .05  High ECSW: *b* = -.50** | Prime X ECSW: *b* = -.03  **Simple effects:**  Low ECSW: *b* = -.10  High ECSW: *b* = -.15 | Prime X ECSW: *b* = -.59*  **Simple effects:**  Low ECSW: *b* = .70^†^  High ECSW: *b* = -.48 | Prime X ECSW: *b* = .30  **Simple effects:**  Low ECSW: *b* = -.91*  High ECSW: *b* = -.47 |

****p*<.001; ***p*<.01; **p*<.05; ^†^*p*<.10

**Study 3: Analysis of DTA**

In study 3, we utilised the DTA task as a way to prime participants. One possibility might be that our death prime was more effective when participants identified more death fragments in comparison to those who found very few (see footnote 9 of the main text). Indeed, the range of scores varied from 1-5 (*M* = 2.87, *SD* = 1.22). Thus, we analysed our mortality salience group only and examined whether individual differences in DTA affected our dependent measures of killing and protection.

For protection, there was a significant DTA X ECSW interaction, *b* = .33, *t* (115) = 4.15, *p* < .001, 95% CI [.17, .49]. The three-way interaction was not significant, *b* = .07, *t* (115) = .80, *p* = .425, 95% CI [-.10, .23]. Simple slopes suggested that at low levels of ECSW, DTA negatively predicted protection, *b* = -.44, *t* (115) = 3.17, *p* =.002, 95% CI [-.71, -.16]. At high levels of ECSW, DTA positively predicted protection, *b* = .33, *t* (115) = 2.27, *p* = .025, 95% CI [.04, .62].

For killing animals, there was also a significant DTA X ECSW interaction, *b* = -.17, *t* (115) = 2.06, *p* = .042, 95% CI [-.34, -.01]. Again, the three-way interaction was not significant, *b* = -.02, *t* (115) = .24, *p* =.808, 95% CI [-.19, .15]. Simple slopes analyses showed that at low levels of ECSW, DTA positively predicted killing albeit not significantly, *b* = .17, *t* (115) = 1.22, *p* = .225, 95% CI [-.11, .46]. At high levels of ECSW, DTA negatively predicted killing animals albeit again this effect did not reach statistical significance, *b* = -.19, *t* (115) = 1.27, *p* = .206, 95% CI [-.49, .11].

1. We opted to compute the scales in line with Study 2 factor solutions as it provided the cleanest and most stable solution. Additionally, the solution of Study 2 made the most sense thematically. Therefore, even though some items did not load, or loaded on a different factor in Study 1 and 3, we opted to disregard this because we felt it would provide a clearer cross-study comparison if the computed scales in each Study were kept consistent. Therefore, there were 5 items (4 in Study 1) for the animal research factor, 5 items (4 in Study 1) in the moral/traditions factor, and 4 items (3 in Study 1) for the animal population factor. [↑](#footnote-ref-1)
